# Cortisol as a Biomarker of Mental Disorder Severity

**DOI:** 10.3390/jcm10215204

**Published:** 2021-11-08

**Authors:** Ewelina Dziurkowska, Marek Wesolowski

**Affiliations:** Department of Analytical Chemistry, Medical University of Gdansk, Gen. J. Hallera 107, 80-416 Gdansk, Poland; marek.wesolowski@gumed.edu.pl

**Keywords:** cortisol, mental disorder, psychotropic drugs

## Abstract

Cortisol—the most important steroid hormone with a significant effect on body metabolism—strongly affects peripheral tissues and the central nervous system. Fluctuations in cortisol secretion often accompany psychiatric disorders, and normalization of its levels correlates with improvement in the patient’s health. This indicates that cortisol may be useful as a biological marker that can help determine the likelihood of mental illness, its impending onset, and the severity of symptoms, which is especially important in the face of the increasing prevalence of mental disorders, including those associated with social isolation and anxiety during the COVID-19 pandemic. This publication reviews recent reports on cortisol levels in healthy participants and shows the current state of knowledge on changes in the levels of this hormone in people at risk for depression, bipolar disorder, and psychosis. It shows how people with psychiatric disorders react to stressful situations and how the applied therapies affect cortisol secretion. The influence of antidepressants and antipsychotics on cortisol levels in healthy people and those with mental disorders is also described. Finally, it reviews publications on the patterns of cortisol secretion in patients in remission.

## 1. Introduction

Mental disorders are one of the most common diseases that can affect anyone. The ongoing pandemic, with the related stress and isolation, has significantly contributed to an increase in the number of cases. Other risk factors for developing mental disorders include genetic factors, perinatal infections, and inadequate nutrition. The World Health Organization (WHO) classifies the following conditions as mental disorders: depression, bipolar disorder, schizophrenia and other psychoses, dementia and developmental disorders, autism [1]. The increasing incidence of mental disorders has generated higher treatment costs, as well as social consequences around those affected. The onset or recurrence of a mental disorder can impair a person’s ability to function at school or work and in everyday social life.

**Figure 1:**
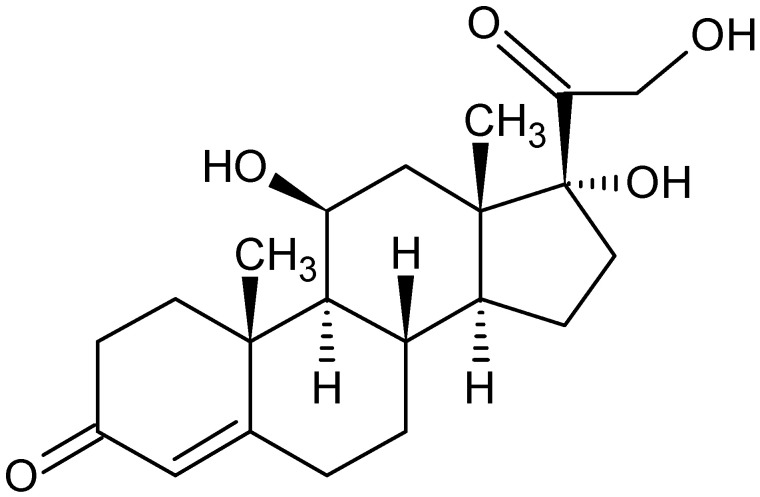
Cortisol structure.

Prolonged exposure to stress (one of the most difficult risk factors to avoid) causes changes in the body, such as an activation of the hypothalamic–pituitary–adrenal axis (HPA), which results in an elevated secretion of cortisol (Figure 1), one of the most important steroid hormones secreted by the adrenal cortex. Cortisol release is regulated by the circadian rhythm and is controlled directly by adrenocorticotropic hormone (ACTH) and indirectly by corticotropin-releasing hormone (CRH). Cortisol reaches its highest blood concentrations between 8 and 10 a.m. [2]. After being synthesized from blood cholesterol, 95% is bound to α_2_ globulin transcortin (corticosteroid-binding globulin, CBG). The limited capacity of transcortin to bind cortisol, results in elevated cortisol levels and a rapid increase in the free form of cortisol in the blood. The action of cortisol occurs via a nuclear receptor stabilized by heat shock proteins (Hsps), of which two Hsp 90 proteins play the most important role.

Due to the ubiquity of glucocorticoid receptors in the body, cortisol (the main glucocorticosteroid hormone) exerts a significant influence on the body’s metabolism, as well as on the functioning of the immune, cardiovascular, skeletal, and nervous systems [3]. Together with insulin and glucagon, this controls glucose homeostasis. Cortisol also influences protein metabolism and the immune system by affecting the concentration, migration, function, and apoptosis of leukocytes. Cortisol also inhibits the secretion of pro-inflammatory mediators such as cytokines and chemokines and reduces the breakdown of mast cells and basophils, which prevents the release of histamine and significantly reduces capillary permeability during an allergic reaction [2].

Due to its structure, cortisol is characterized by strong lipophilicity, which allows it to penetrate into the central nervous system, through the site of two types of corticosteroid receptors with an affinity for cortisol: glucocorticosteroid receptors (GR), located in the hippocampus and prefrontal cortex, and mineralocorticosteroid receptors (MR) located evenly throughout the central nervous system. Mineralocorticosteroid receptors have a stronger affinity for cortisol, such that they are saturated to about 90% regardless of the level of cortisol secretion in the body, compared to 10% saturation of glucocorticosteroid receptors, which can increase to 90% in stressful situations when cortisol secretion increases or when cortisol concentrations peak during the circadian cycle [4]. Stimulation of each type of receptors elicits a different response from the body. Activation of the mineralocorticosteroid receptors is essential during the first phase of a stressful stimulus and is also responsible for maintaining the basal activity of the HPA axis. In contrast, activation of glucocorticosteroid receptors under physiological conditions inhibits neuronal excitability, which extinguishes the stress response [4]. Under physiological conditions, changes in cortisol concentrations determine the appropriate response of the body to stress. However, persistently high cortisol concentrations over a long period of time may have toxic effects, leading not only to metabolic changes such as diabetes, osteoporosis, or muscle cachexia but also to changes in the central nervous system [2].

Many activities that cause an increase in cortisol attest to its important role in the body. Both its deficiency and excess will have specific consequences that often relate to mental disorders. Therefore, the aim of the following paper is to systematize information on HPA axis dysregulation and the influence of cortisol levels on the possibility of developing a mental disorder, such as depression, bipolar disorder (BD), or an episode of psychosis. Moreover, this work presents changes in the level of cortisol during the course of the disease, as well as the influence of the applied therapy on levels of the hormone.

## 2. Consequences of Abnormal Cortisol Secretion

Under physiological conditions and in a stress-free situation, healthy adults secrete between 10 and 20 mg of cortisol in the daily rhythm [2]. This changes significantly in stressful situations and when the body’s circadian rhythm is disturbed, e.g., when the sleep–wake rhythm is disrupted. The recovery of cortisol secretion is relatively slow; moreover, glucocorticosteroids alter the expression of biological clock genes in the kidneys, lungs, and muscles, which may further slowdown re-synchronization of the circadian clock [3].

Glucocorticoids (such as cortisol) readily penetrate into the brain. Although their physiological role in the functioning of the central nervous system is unknown, it is assumed that their normal concentration ensures adequate neuroplasticity, growth, and differentiation of neurons, and abnormal concentrations can affect both behavior and cognitive functioning. Initially, excessive levels of cortisol cause euphoria, but prolonged exposure of the brain to a high concentration can result in the appearance of other psychological symptoms such as irritability, emotional lability, and depression. Cortisol deficiency may also produce irritability, but in this case, most patients are apathetic and depressed [5,6].

Disorders in cortisol secretion (particularly hypercortisolemia) may cause mental disorders and can be one of the many hormonal disorders accompanying these conditions, for example depression. About 50% percent of patients with newly diagnosed depression have been observed to have excessive cortisol secretion [7]. It is important to remember that depression is accompanied not only by the dysregulation of the HPA axis but also by abnormal thyroid function or sex hormone dysregulation during the postpartum and postmenopausal periods [8].

Increased secretion of cortisol in a stressful situation has consequences for the functioning and condition of our brain. The structure most exposed to high concentrations of this hormone is the hippocampus due to its large number of steroid receptors [9]. Recurrent stress causes changes in the structure of neurons. When stress is short lived, the onset of atrophy is reversible, but long-term stress can lead to the death of neurons located in the hippocampus. PET (positron emission tomography) and fMRI (functional magnetic resonance imaging) studies indicate that, in the case of diseases such as depression or post-traumatic stress disorder, there is a reduction in the volume of this region of the brain, as well as in that of the prefrontal cortex and amygdala [10]. It is not certain, however, whether the increase in cortisol secretion itself or the dysregulation of cortisol secretion are the cause of atrophy. In addition, a reduction in hippocampal volume is also seen in borderline personality disorder [10] and patients with schizophrenia [11].

The level of cortisol is most often measured in the blood, in which its total concentration is determined. When testing the level of this hormone in other biological material, such as saliva, urine, or hair, only the free fraction, i.e., the one not bound to proteins, is determined. Blood, saliva, and urine allow the determination of the current level of the hormone; but when it is advisable to test over a month or longer, the analysis is carried out using hair, in which cortisol accumulates, and the average growth rate is 1 cm per month.

In the case of biological material, the time of sample collection should be taken into account. Cortisol can be measured in the morning, afternoon, and evening hours. As mentioned, the secretion of cortisol is cyclical, and the highest concentration is recorded in the morning. Observation of the morning cortisol awakening response (CAR) may indicate mental disorders, due to the fact that it may have a different course in healthy people and in those with mental disorders. For this purpose, several samples are taken, most often four at certain intervals. In the event that one sample of the material for testing is taken, it can be done in the afternoon or in the evening. The first possibility is to measure the concentration of a hormone while its levels are relatively in equilibrium. When using option two, should be noted that evening cortisol level is the lowest. Therefore, an appropriately sensitive analytical method should be selected that enables the determination of the hormone in biological material.

In some cases, determination of cortisol levels is preceded by a test with the exogenous orally administered corticosteroid (DEX), which strongly inhibits the secretion of endogenous corticotropin. Inhibition of CRH secretion leads to a decrease in the level of ACTH and, consequently, also of cortisol. The DEX test is used in the diagnosis of Cushing’s syndrome and depression. It is also possible to use a modified DEX/CRH test. During this test, a few hours after oral administration of dexamethasone and sampling for analysis, human corticoliberin is administered subcutaneously for stimulation of ACTH secretion and consequently cortisol. This test is used in the diagnosis of depression [12].

## 3. The Role of Cortisol in the Pathophysiology of Depression

Depression is one of the most common mental diseases in the world. It is a complex and multifactorial disease, most frequently manifested by a depressed mood and reduced psychomotor drive. Additionally, it may be accompanied by circadian rhythm disorders (e.g., sleep disorders) as well as anxiety, pain, weight fluctuations, and other somatic symptoms. Patients often have suicidal thoughts, feelings of guilt, and loss of interest. Depression may also accompany other conditions, such as cardiovascular disease, cancer, hormonal disorders, viral infections, and vitamin deficiencies [1,13].

There are several mutually complementary hypotheses on the etiology of depression. The oldest monoaminergic theory is based on observing the mechanism of action of two of the first tricyclic antidepressants (TCAs), imipramine and iproniazid, which produce adaptive changes in the density and reactivity of the adrenergic, serotonin, and dopaminergic receptors [4].

Genetic factors and dysregulation of the immune system activity have also been implicated in the pathogenesis of depression. The genetic transmission of depression is weaker than that for bipolar disorder, but unipolar depression has been found to be more common in monozygotic than in dizygotic twins. The role of the immune system in inducing depression is associated with an increased secretion of certain pro-inflammatory cytokines [13].

Depression is also associated with neuroendocrine disorders. The most significant of these include disturbances in the secretion of cortisol, corticotropin (ACTH), and corticotropin-releasing hormone (CRH), as well as an impaired feedback mechanism inhibiting the secretion of CRH. Thyroid hormone release is also dysregulated due to an impaired response of thyrotropic hormone (TSH) to the action of the parent hormone—thyroxine (TRH) [13].

The aforementioned hypotheses for the pathophysiology of depression are complementary, because the neuroendocrine, monoaminergic, and neurotrophic systems are closely related. Dysregulation of the HPA axis contributes to a suppression of transcription of the brain-derived neurotrophic factor (BDNF) gene, a protein that belongs to the nerve growth factor family. As mentioned above, glycoprotein receptors are abundantly distributed in the brain, particularly in the hippocampus. Long-term exposure of the cortisol receptors, observed during severe stress as well as major depression, results in decreased synthesis and secretion of BDNF. As a result, neurodegenerative changes are observed in some brain structures, primarily in the hippocampus [8].

A chronic excess of cortisol in the brain may also lead to serotonin (5-HT) deficiency due to the decreased availability of tryptophan—the substrate for 5-HT production. Moreover, it reduces the density and reactivity of serotonin receptors [4]. It is presumed that currently used antidepressants have the opposite effect to stress by increasing the secretion of BDNF. Additionally, they not only affect neurotransmitters and activate amine receptors but also normalize the activity of the HPA axis, decreasing the levels of CRH, and consequently also ACTH and cortisol [8].

One weaknesses of the monoaminergic hypothesis is the fact that the administration of drugs that increase the levels of monoamines gives noticeable clinical effects only after several weeks, while often patients require immediate help. The explanation for this phenomenon may be adaptive changes of the second messenger, as well as a change in the activity of transcription factors, for example concerning the synthesis of neurotrophic factors. It is assumed that the time required for the synthesis of proteins, e.g., BDNF, is usually 2 weeks or even longer, which coincides with the time of improvement in the patient’s condition [8,13].

The significant influence of cortisol on the body’s metabolism, gene expression, and the central nervous system causes a dysregulation of the HPA axis (and consequently fluctuations in cortisol levels), which strongly affects the mental state of patients. Therefore, cortisol is considered one of the most significant biomarkers of anxiety disorders and depression [14]. While the exact mechanism of the normalization of cortisol secretion in depression is not fully understood, recognizing whether the cessation of symptoms correlates with a decrease in the hormone may support whether cortisol levels may be used as a marker of undiagnosed depression [15]. A study by Fiksdal et al. [15] showed that individuals not diagnosed for depression who exhibited symptoms of the illness during the Trier Social Stress Test (TSST) showed a strong response to social stress in the form of an increase in cortisol secretion. They also found that the HPA axis response resulting from anxiety and depression symptoms differed in terms of response to and recovery from the psychosocial stressor. The anxiety symptoms were characterized by a blunted response to and recovery from the stressor. In contrast, both curves were steeper in the case of depression. It was especially noticeable in the case of women, who reacted more strongly with the secretion of cortisol [15]. This may result in rapid dysregulation of the HPA axis and the appearance of clinical symptoms of depression. A question also arises whether the increase in cortisol secretion is correlated with the gender of the patient. 

A study was conducted to determine how gender would affect the stress-related response caused by conflict with a partner. Then the observations were related to the diagnosed depression or anxiety disorder. The study showed that regardless of gender, there was an increase in cortisol secretion. However, in the case of women, the increase in cortisol secretion was more correlated with a diagnosis of anxiety disorders than with a diagnosis of depression. In the case of both diagnoses in men, there was an increase in cortisol secretion, which correlated with the severity of symptoms [16].

Elevated cortisol secretion may contribute to central nervous system damage [8]. Therefore, the next question is whether elevated cortisol levels are associated with cognitive impairment, as often observed in people with mental disorders. Høifødt et al. [17] studied cortisol levels in patients treated for depression and in those with a history of depressive episodes. Compared to controls without a history of depressive episodes, the patients with depression had elevated levels of cortisol, which strongly correlated with the severity of symptoms and occurred only in the evening. Such a correlation was not observed in patients with a history of depressive episodes, which may indicate normalization of HPA during disease remission. Both study groups did show weaker cognitive abilities than healthy individuals, which in turn may indicate that the impairment of cognitive functions was not directly related to cortisol levels [17]. Despite this, impaired cognitive performance and decreased verbal memory have been observed in individuals with psychotic major depression and schizophrenia spectrum disorders, where elevated cortisol levels have been confirmed relative to the controls [18].

Finally, depression is a condition that can occur regardless of patient age, where the severity and resistance to treatment are also independent of age. A study was conducted to determine whether there was a relationship between depression treatment’s progress with cognitive behavioral therapy and cortisol levels [19], which showed that older adults who had high cortisol levels were less likely to respond to this therapy, an observation with important implications for treatment selection and prognosis.

Previous studies show that in the case of depression and anxiety symptoms, the level of cortisol increases. TSST is very often used to determine the activity of the HPA axis. On the basis of the presented studies, it can be concluded that, in the case of depression and anxiety symptoms, untreated patients reacted differently to stressful situations depending on the type of disorder, when the course of the stress response and recovery curves was different. Apart from the disease itself, gender also determined the course of the studied curves. The studies also show that during remission of the disorder, the activity of the HPA axis was normalized. However, despite the remission and normalization of cortisol secretion, cognitive dysfunction still remained in patients with depression or a depressive episode in the past. It may be associated not only with changes in the brain that occur during the disorder but also with the age of the patients.

## 4. Cortisol Secretion Disorders and Bipolar Disorder

Abnormal cortisol secretion is often reported during depression. Although it is not treated as a determinant of the illness, it can often hinder and complicate treatment. Importantly, cortisol levels depend on the type and phase of a depressive disorder. In bipolar disorder (BD), an increase in cortisol secretion may be seen in the manic phase [20]. Another study showed that high cortisol awakening response (CAR) was present in both unipolar depression and bipolar disorder, but both conditions differed in the course of the changes in the daily curve of the hormone concentration, which was evidently influenced by manic symptoms [21]. Huang et al. examined hormone levels in patients during depression, mania, and partial remission against those of healthy control subjects [22]. Regardless of the patient’s condition, a weaker cortisol awakening response was observed, which confirms dysregulation of the HPA axis in BD subjects, although the course of the cortisol curve itself varied slightly depending on the phase of the disease. 

As bipolar disorder is characterized by significant genetic transmission [13], cortisol levels are being studied in the offspring of patients with bipolar disorder. In one experiment, genetically burdened children were found to have higher levels of cortisol both in the morning and during the day. Additionally, it was noted that no child showed symptoms of BD at the time of the study, which may indicate that HPA axis dysregulation is an early anomaly that precedes the onset of other symptoms [23]. However, these results were undermined by a subsequent study performed on a large group of genetically burdened adolescent children of parents who were in remission, which showed normal cortisol levels [24], indicating normalization of the HPA axis, similar to an analogous situation with parents with unipolar depression [17]. This was confirmed by another study where cortisol levels were measured in at-risk offspring in the morning, during the day, and in the evening. No significant changes in hormone secretion were seen in the children of parents with BD and healthy controls. Interestingly, when the results were analyzed within the study groups, a large interindividual variability in cortisol levels was observed among the children in the risk group [25].

Cortisol as an indicator of the genetic burden of BD was also used during sibling studies. Determination of the hormone was performed in subjects with bipolar disorder in remission and healthy siblings. The results obtained were compared with those of the control group. An impaired response to the Trier Social Stress Test (TSST) was observed, in the form of lower cortisol levels in subjects in remission. However, there were no differences in HPA axis activity in healthy siblings and in the control group, which excluded the risk of familial bipolar disorder. On the other hand, blunted HPA axis activity and weaker cortisol secretion in stressful situations are due to the effects of the drug therapy [26].

Cortisol levels have been tested in patients with BD with suicidal behaviors [27,28]. The first study examined cortisol levels without exposing patients to stress [27]. Results showed elevated levels of the hormone both measured upon awakening and in the evening. For patients with BD who did not exhibit suicidal behavior, cortisol levels were comparable to those in the control group. In contrast, when HPA axis activity in response to stress was examined, lower cortisol levels were observed in individuals after suicide attempts, which may be an indicator of this type of behavior in patients with BD [28].

In summary, CAR was used for the most common HPA axis activity tests in patients with BD. A weaker response of the HPA axis to awakening was then observed in people with BD. As in depression, so also in BD, the remission normalization of cortisol secretion was observed. An interesting issue concerning BD is the issue of genetic transmission. One of the studies showed such a situation, when higher cortisol levels were obtained in children of BD patients, but subsequent studies did not confirm this theory. The risk of familial BD was also excluded, but in this case, the study was conducted using TSST.

## 5. Cortisol Secretion Disorders and Psychosis

HPA axis dysregulation and associated cortisol secretion disorders are most commonly observed in depression as well as in BD. It has also been noted that stress, which causes an increase in cortisol levels, may contribute to the relapse of not only depression or BD but also schizophrenia [18,20]. On the other hand, the elevated cortisol levels can lead to psychiatric disorders [8]. It was observed in women during the first episode of psychosis, who had elevated cortisol levels correlated with the self-reflection process [29]. Therefore, the question should be asked whether cortisol can be a determinant of the state of a patient at risk for psychoses, for example. Recent studies have attempted to answer this question.

Walker et al. studied young adults who met the criteria for psychosis risk and who had their cortisol levels measured several times over the course of a year to monitor for psychosis [30]. Compared with the not-at-risk control group, individuals who developed psychosis had significantly higher cortisol levels [30]. Similar conclusions were reached by Labad et al., who observed elevated levels of cortisol measured upon awakening in individuals at risk of developing psychosis [31]. Disturbed secretion of this hormone was particularly evident in patients not treated with antipsychotics.

Elevated cortisol levels appear to be correlated with the risk of a first psychotic episode [32], but symptom severity is only correlated with cortisol levels during the initial phase of psychosis [33,34,35], particularly with positive psychotic symptoms [33] or increased anxiety [35]. The observed increase in cortisol secretion is particularly evident during morning measurements [34]. 

The daily concentration of cortisol was observed to be higher in children with behaviors that may indicate the onset of schizophrenia and in children with a family history of schizophrenia. In addition, in children at genetic risk, the daily cortisol curve was blunted, which indicates impairment of the HPA axis function. On the basis of the obtained results, the authors assume that the HPA dysfunction in the form of a blunted cortisol awakening response is a factor of susceptibility to psychosis, while daily elevated cortisol concentration is a factor preceding the onset of the disease [36].

The onset of psychosis can be the beginning of schizophrenia. As was the case in BD, here also stress can cause relapse or exacerbation of symptoms of the disease. Therefore, in schizophrenia as well, examination of cortisol levels may indicate abnormal functioning of the HPA axis in the form of a blunted cortisol awakening response [37]. In both diseases, sensitivity to perceived stress is very high, which may further contribute to the relapse. However, cortisol levels in BD are higher than in schizophrenia, particularly in hospitalized patients. In both schizophrenia and BD, cortisol levels correlate with the symptoms of the disorder [38]. Studies also suggest that patients with paranoid schizophrenia have hypersensitivity of dopamine receptors in the hypothalamus, resulting in increased HPA axis activity. In contrast, in BD depression, increased HPA axis activity is associated with thyroxine (TRH) dysfunction, which may explain the differences in stress response between patients with schizophrenic and BD [39]. Elevated cortisol levels are also observed in patients with borderline personality disorder [40]. This disorder is difficult to diagnose and often inadequately treated, and in many cases leads to suicide. 

Previous studies indicate that the first episode of psychosis may be preceded by an increase in cortisol secretion. In addition, the course of CAR in this case may differ from the course of the curve of healthy people. It is most often observed that the cortisol curve is then blunted. Research also shows that cortisol levels correlate with the symptoms.

A summary of information on cortisol levels in people with mental disorders is presented in Table 1.

## 6. Psychotropic Drugs and Cortisol Secretion

Previous research indicates that HPA axis abnormalities accompany many mental disorders [16,17,18,19,20,21,22,27,28,29,30,31,32,33,34,35,36,37,38,39,40]. Changes in cortisol secretion may indicate impending illness, as is the case with psychosis. Conversely, concentrations of secreted cortisol may indicate the severity of the illness, as observed in depression. Therefore, it is worth examining how psychotropic drugs can affect cortisol levels. Will the secretion of the hormone be normalized under their influence, and will it be proportional to the improvement of the patient’s condition?

### 6.1. The Effect of Antidepressants on Cortisol Levels

Antidepressants are the most commonly studied drugs in terms of their effects on cortisol secretion. They are characterized by great diversity in both chemical structure and their mechanisms of action, resulting in different effects on the HPA axis. Stabilization of their activity may result from a direct action on CRH receptors, resulting in the inhibition of corticoliberin secretion and subsequently a decrease in ACTH and cortisol secretion. Some of these drugs, such as tricyclic antidepressants (TCAs), affect GR receptor biosynthesis by increasing GR mRNA levels [41]. In this way, they stimulate the inhibitory feedback mechanism that is weakened during depression.

The varying effects of antidepressants on cortisol levels are also due to the health status of the study participants. It has been repeatedly pointed out that some antidepressants can increase cortisol levels in healthy volunteers. An example of such a drug is citalopram (serotonin reuptake inhibitor, SSRI), which not only increases the secretion of cortisol [42,43] but also acts proportionally to the applied dose [42]. It has been shown that this effect was achieved by the drug’s influence on several serotonergic (5-HT) receptor subtypes and not only on the 5-HT_2A_ receptor as previously thought. In contrast, reboxetine, which inhibits norepinephrine reuptake, does not affect cortisol levels in healthy individuals [43]. Venlafaxine, which inhibits secondary reuptake of both serotonin and norepinephrine, affects the HPA axis via serotonin receptors and, just like citalopram, causes an increase in cortisol levels [44,45]. Its effect is also proportional to the dose used [44]. Similarly, in a stressful situation, cortisol levels in healthy volunteers are influenced by tianeptine, which, despite its inhibitory effect on serotonin reuptake, also causes an increase in cortisol secretion [46].

The various effects of antidepressants on study volunteers are not only due to the group of the drug used. For example, escitalopram, which is the S-enantiomer of citalopram, can reduce cortisol levels in healthy subjects exposed to stress [47]. In contrast, antipsychotics characterized by a different mechanism of action normalize the HPA axis in a different way than antidepressants. Haloperidol, acting through a blockade of dopamine D_2_ receptors, normalizes the HPA axis by acting antagonistically through the same receptors. This action is so strong that, in healthy individuals, after the administration of haloperidol, a strong decrease in cortisol secretion is observed, which was not observed after the administration of aripiprazole [48]. When examining the effect of quetiapine and olanzapine (atypical antipsychotics) on the HPA axis, it was noted that their strong influence is determined mainly by the blockade of serotonergic receptors and, to a lesser extent, adrenergic and histamine receptors. The effect of these drugs was to reduce cortisol levels in healthy subjects [49].

As mentioned above, antidepressants, in addition to acting on neurotransmitters and amine receptors, also normalize HPA axis activity [8]. Studies in which patients with depression were treated with tricyclic antidepressants such as amitriptyline have shown a reduction in cortisol levels; however, complete normalization usually occurs after several weeks of normalization of HPA axis activity and reduction in hormone levels [50,51,52,53]. As mentioned above, long-term high cortisol levels in the brain contribute to neurodegenerative changes that can be observed by the positron emission method [54]. Simultaneously, cortisol levels were determined before and after treatment with TCAs (amitriptyline), SSRIs (fluvoxamine), and SSA (mianserin). This showed that brain abnormalities, particularly in the limbic and paralimbic areas, resolved as a result of the treatment, with the exception of lesions in the prefrontal cortex. In addition, cortisol levels were reduced regardless of the therapy used.

When comparing the effectiveness of normalization of the HPA axis activity by TCA and SSRI therapy, it was observed that the less selective old-generation drugs cause stronger changes in cortisol secretion, which is manifested not only by a decrease in daily cortisol secretion but also by a blunted cortisol awakening response [55]. 

SSRIs are among the most commonly administered drugs for the treatment of depression. It has already been noted above [42,43,44,45] that their effect on HPA axis activity is highly dependent on the health status of the volunteer. In the case of patients with depression, citalopram has been found to decrease cortisol levels [56,57,58]. Moreover, the reduced cortisol secretion is closely dependent on the concentration of citalopram in the biological material studied. Therefore, it is advisable to determine its normalizing effect on HPA axis activity based on the concentration of the substance in the biological material and not solely on the dose used [56]. The normalization of HPA axis activity in the case of citalopram results from direct effects on the adrenal cortex [58] and the serotonin system, and the therapeutic effect achieved is correlated with cortisol levels [57].

Escitalopram also affects the number and sensitivity of serotonin 5-HT_1A_ receptors in a similar way to citalopram, but not all studies have confirmed its effect on cortisol secretion levels. In one of them, no reduction in the level of the secreted hormone was observed, although an improvement in patient condition was observed [59]. It should be noted, however, that no increase in cortisol secretion was observed in patients after escitalopram therapy during the study. Other conclusions were reached by Lenze et al., who not only found normalization of cortisol secretion under the influence of the applied treatment but also noted improvements in immediate and delayed memory [60]. Moreover, memory improvement was strongly correlated with a decrease in cortisol levels [60].

As already mentioned, SSRIs are most likely to reduce cortisol levels in patients with depression, but this requires treatment over several weeks, and the effects are often more pronounced in severely depressed patients, late responders, and non-responders [8,61,62,63]. This was also confirmed by determining the effect of sertraline on hormone levels, before and after a four-month treatment with the drug [64]. Paroxetine, on the other hand, decreased waking cortisol levels but did not affect cortisol awakening response. Furthermore, the drug’s effect on hormone levels was more pronounced in late responders and non-responders and only after 12 weeks of therapy [65].

Venlafaxine, similar to other drugs from the SSRI group, has a varying effect on cortisol secretion depending on the health status of the volunteers. In patients with depression with elevated cortisol levels, the drug reduces blood cortisol after several weeks of therapy [66,67]; however, there was no full normalization of HPA axis activity, as manifested by elevated ACTH levels [67]. During other studies on the effect of venlafaxine on cortisol levels, it was observed that in the course of depression there is a decrease in the activity of enzymes responsible for the breakdown of cortisol. It was observed that during therapy, venlafaxine not only normalized cortisol levels but also influenced cortisol metabolism by increasing the bioavailability of cortisol in tissues and activating the enzymes that break it down. Similar effects on hormone metabolism have been observed during mirtazapine treatment [68].

Mirtazapine, which acts antagonistically to adrenergic and serotonin receptors, decreases hormone secretion in healthy volunteers. However, in the case of patients with depression, the effect of mirtazapine is not so clear. In most described studies, after several weeks of mirtazapine therapy, the HPA axis activity was normalized, and cortisol levels decreased [69,70,71]. Other studies indicate that after the first week of treatment there was a decrease in cortisol levels, but after about the fifth week of therapy, the cortisol levels increased slightly, although the increase was no longer as high as at the beginning of treatment [72]. In the same study, it was observed that reboxetine decreased cortisol secretion more slowly but steadily, so that in just the fifth week of therapy, the hormone level corresponded to the value in healthy subjects [72].

Among the antidepressants, tianeptine is the only compound that acts by directly inhibiting the HPA axis, thereby increasing neuronal plasticity. Moreover, unlike other antidepressants, it increases serotonin reuptake. Due to its mechanism of action, it should strongly decrease cortisol secretion. However, earlier studies have indicated that its effect on cortisol levels is insignificant [73].

### 6.2. The Effect of Combined Therapy on Cortisol Levels

Studies that compare the effects of various groups of psychotropic drugs on cortisol levels are most often concerned with comparing the patient’s response to the applied therapy, i.e., a change in cortisol levels and improvement in their condition. It was mentioned above that for the at-risk group, elevated cortisol levels can cause psychosis [32,33,34,35]. Sugranyes et al. showed that people who are at risk for psychosis and untreated have higher cortisol levels than those in the control group and those at risk for psychosis but receiving treatment. Treatment of at-risk individuals with antidepressants (SSRIs) or atypical antipsychotics resulted in improved patient status and lower cortisol secretion [74]. Another study comparing the effects of escitalopram and quetiapine in monotherapy (escitalopram) and combination therapy on cortisol levels in patients treated for depression, found that the reduction in hormone levels was more pronounced in combination therapy, which may be reflected in patient improvement [75]. Typically, the use of combination therapy results in better outcomes when treating drug-resistant depression [53].

### 6.3. The Effect of Antipsychotics on Cortisol Levels

The normalizing effect of atypical antipsychotics (quetiapine, risperidone, aripiprazole, haloperidol, and olanzapine) on HPA axis activity has also been studied in patients during their first episode of psychosis [76]. The study showed that patients treated for less than 14 days had higher cortisol levels than those treated for more than 2 weeks. In addition, patients showed a blunted cortisol awakening response compared to the control group. The study confirmed the effect of the antipsychotic drugs on circadian cortisol secretion, but they did not affect cortisol awakening response.

Atypical antipsychotics are most commonly used to treat schizophrenia. As already mentioned, changes in HPA axis activity are also observed in schizophrenia. Studies on changes in cortisol secretion during therapy with risperidone or haloperidol in patients with schizophrenia confirmed that higher cortisol levels were correlated with negative symptoms in the case of schizophrenia, while normalization of cortisol secretion and relief of symptoms of the disease were observed after 12 weeks of treatment with both risperidone and haloperidol, with the atypical antipsychotic drug contributing to a stronger decrease in cortisol [77]. A summary of information on the effect of psychotropic drugs on cortisol levels is presented in Table 2

## 7. Conclusions

Cortisol is a basic steroid hormone with secretion disturbances caused by not only metabolic but also mental changes, and so it can be a valuable source of information about a patient’s mental state. The studies conducted so far indicate that an increase in cortisol secretion is observed during many mental disorders, such as depression, BD, or schizophrenia. However, the applied therapy normalizes the activity of the HPA axis. In most cases, a decrease in cortisol levels also correlates with improvement in the patient’s condition. Moreover, studies indicate that cortisol determination may be helpful in predicting the effectiveness of a therapy.

In everyday life, the observed increase in cortisol levels may be a physiological response to stress [2]. In addition, the normalization of its secretion during the therapy used to treat mental illness proves remission [56,57,58,75]. However, long-term exposure to high levels of cortisol in the most sensitive brain structures, such as the hippocampus, may result in neurodegenerative changes as well as failure to respond to treatment.

However, using cortisol as an auxiliary determinant of the patient’s health status and the effectiveness of the therapy applied, one should first pay attention to the time of sampling and the type of test performed, which must be selected according to the type of disorder.

In the case of depression, test samples are most often taken in the afternoon when the cortisol level is constant. Very often, the activity of the HPA axis is determined on the basis of TSST. Samples are taken in the morning less frequently due to too large a change in cortisol at the beginning of the day. On the other hand, when examining the level of cortisol in people with BD or a psychotic episode, CAR is defined. In the case of BD, the level of cortisol was also tested in the evening hours. However, when determining the effect of pharmacotherapy on the secretion of cortisol, samples were taken most often before the start of therapy and after its completion. The most common materials for testing the level of cortisol in both untreated patients and in those undergoing psychotropic drug therapy are saliva and blood.

## Figures and Tables

**Table 1:** Disorders of cortisol secretion in untreated patients.

The Patient’s Condition	Terms of Sampling	Cortisol Secretion	Additional Insights	Ref.
Undiagnosed depression	saliva/afternoon before and after TSST	↑	-	[11]
Depression	saliva/afternoon	↑	-	[13]
Anxiety disorders				
Depression in remission	saliva/morning, evening	N	cognitive decline	[14]
Depression		↑		
Schizophrenia spectrum disorders	blood/afternoon	↑	cognitive decline, verbal memory decline	[15]
Psychotic major depression				
Elderly depressed	saliva/afternoon	↑	less responsive to cognitive behavioral therapy	[16]
Bipolar disorder	-	↑	increase in cortisol secretion associated with the manic phase	[17] review
Unipolar depression	saliva/CAR, evening	↑	elevated CAR	[18]
Bipolar disorder				
Bipolar disorder	saliva/CAR	↑	elevated CAR	[19]
Offspring of parents with bipolar disorder	saliva/CAR, bedtime	↑	HPA axis disorders as an early BD anomaly	[20]
Offspring of parents with bipolar disorder in remission	saliva/CAR, evening	N	HPA axis normalization in remission	[21,22]
Bipolar disorder in remission	saliva/afternoon	N	weaker response to TSST	[23]
Siblings of person with bipolar disorder in remission		N	no family risk of bipolar disorder	
Suicide attempts in bipolar disorder	saliva/awakening, bedtime	↑	weaker response to stress after a suicide attempt	[24]
	saliva/various times of day			[25]
People at risk of developing psychosis	saliva/3 times a day	↑	-	[27]
	blood/morning saliva/CAR			[28]
First-episode psychosis	-	↑	cortisol–symptoms correlation only in the initial phase of psychosis	[29] review
	saliva/morning		cortisol–symptoms correlation with positive symptoms	[30]
	saliva/morning		cortisol–symptoms correlation with severe anxiety	[32]
	saliva/CAR		-	[31]
Children at elevated risk for schizophrenia	saliva/CAR	↑	blunted CAR	[33]
First-episode schizophrenia	saliva/6 times after TSST	↑	blunted CAR	[34]
Borderline personality disorder	saliva/before and after TSST	↑	-	[37]

↑—increase in cortisol concentration; N—normal cortisol concentration; CAR—cortisol awakening response; TSST—Trier Social Stress Test; HPA—hypothalamic–pituitary–adrenal axis.

**Table 2:** Effect of the applied therapy on the cortisol secretion.

The Patient’s Condition	Pharmacological Intervention	Biological Matrix	Cortisol Secretion	Additional Insights	Ref.
Healthy volunteers	citalopram	blood	↑	increase in cortisol concentration proportional to the applied dose	[39]
		saliva			[40]
	reboxetine	saliva	NI	-	[40]
	venlafaxine	blood	↑	increase in cortisol concentration proportional to the applied dose	[41]
		saliva		-	[42]
	tianeptine	blood	↑	-	[43]
	escitalopram	saliva	↓	stressful situation	[44]
	haloperidol	saliva	↓	-	[45]
	aripiprazole		NI		
	quetiapine/olanzapine	blood	↓	-	[46]
Depression	amitriptyline	blood	↓	normalization occurs after a few weeks of therapy	[47,49]
		saliva			[48,50]
	amitriptyline/fluvoxamine/mianserine	blood	↓	reduction of anomalies in the brain, in particular, in the limbic and paralimbic areas	[51]
	citalopram	blood	↓	reduction of cortisol concentration proportional to the administered dose	[53]
		blood		therapeutic effect proportional to the concentration of cortisol	[54]
		blood		-	[55]
	escitalopram	saliva	NI	-	[56]
	escitalopram	saliva	↓	improvement of immediate and delayed memory correlated with the concentration of cortisol	[57]
	SSRI	saliva	↓	effects visible in people with severe depression, late responders, or non-responders	[58,59,60]
	sertraline	saliva	↓	-	[61]
	paroxetine	saliva	↓/NI	effects seen in late responders and non-responders/no effect on CAR	[62]
	venlafaxine	saliva	↓	-	[63]
		saliva		lack of full normalization of HPA axis functions	[64]
	venlafaxine/mirtazapine	urine	↓	increasing the bioavailability of cortisol in tissues and activation of degrading enzymes	[65]
	mirtazapine	saliva	↓	-	[66]
					[68]
		blood			[67]
	mirtazapine	blood	↓/↑	decrease in cortisol level after the first week of treatment, slight increase after the fifth week of treatment	[69]
	reboxetine		↓	lowering cortisol levels in the fifth week of therapy	
	escitalopram, escitalopram + quetiapine	blood	↓	stronger reduction of the cortisol level in combination therapy	[72]
People at risk of developing psychosis	antipsychotics	saliva	↓	-	[71]
	SSRI				
First-episode psychosis	quetiapine/risperidone/aripiprazole/haloperidol/olanzapine	saliva	↓	blunted CAR in patients	[73]
Schizophrenia	risperidone/haloperidol	blood	↓	-	[74]

↑—increase in cortisol concentration; ↓—decrease in cortisol concentration; NI—no impact; SSRI—selective serotonin reuptake inhibitor.

## Data Availability

Not applicable.
